# Leukemia Stem Cell Frequency at Diagnosis Correlates With Measurable/Minimal Residual Disease and Impacts Survival in Adult Acute Myeloid Leukemia

**DOI:** 10.3389/fonc.2022.867684

**Published:** 2022-04-08

**Authors:** Azza M. Kamel, Nahla M. Elsharkawy, Eman Z. Kandeel, Marwa Hanafi, Mohammed Samra, Randa A. Osman

**Affiliations:** ^1^ Department of Clinical Pathology, National Cancer Institute, Cairo University, Cairo, Egypt; ^2^ Department of Medical Oncology, National Cancer Institute, Cairo University, Cairo, Egypt

**Keywords:** AML, LSC, MRD, CD123, CD133

## Abstract

Acute myeloid leukemia (AML) is a heterogenous disease in which the initiation and maintenance of the malignant clone is blamed on a rare population of leukemia stem cells (LSCs). The persistence of such a malignant population is referred to as measurable/minimal residual disease (MRD). Evaluation of MRD is the gold standard for follow-up of therapy and constitutes an independent prognostic parameter. As LSCs are the main contributor to the persistence of MRD, then MRD should correlate with the bulk of LSCs at the individual case level. MRD is measured at defined time points during therapy. However, LSCs can be evaluated at diagnosis, which ensures the advantage of early prediction of high-risk patients and allows for early therapeutic decisions. Using two simple four-color monoclonal antibody combinations (CD38/CD123/CD34/CD45 and CD90/CD133/CD45/CD33) and the prism function of the Coulter Navios flow cytometer, the frequency of LSC subsets was evaluated in 84 newly diagnosed adult AML patients. For each panel, 16 possible combinations were detected. Our results showed that there was extreme variability in the percentage of the LSC fraction between different cases, as well as at the individual case level. For each LSC subset, the median value was used to divide cases into low and high expressors. LSC subsets that showed an impact on overall survival (OS) and disease-free survival (DFS) included CD123+, CD 123+/CD34-, CD34-/CD38+/CD123+, CD34+/CD38-/CD123+, CD133+, and CD133+/CD33-. On multivariate analysis, only CD123 (p ≤ 0.001, SE = 0.266, HR = 2.8, 95% CI = 1.74.7) and CD133+/CD33- (p = 0.017, SE = 0.263, HR = 1.9, 95% CI = 1.1–3.1) retained their significance for OS. Likewise, only CD34+/CD38-/CD123+ (p ≤ 0.001, HR 2.3, SE: 0.499, 95% CI: 2.4–17.4) and CD133 (p = 0.015, HR 2.3, SE 0.34, 95% CI: 1.2–4.4) retained their statistical significance for DFS. The LSC frequency at diagnosis showed a moderate to strong correlation with MRD status at day 14 and day 28. In conclusion, the level of LSCs at diagnosis correlated with MRD status at day 14 and day 28 in AML patients and had a deleterious impact on OS and DFS. It may be used as an early marker for high-risk patients allowing for early therapeutic decisions.

## 1 Introduction

Acute myeloid leukemia (AML) is an extremely heterogeneous hematological malignant disease. Classification of AML integrates morphological, cytochemical, immunophenotypic, and molecular genetic characteristics; all these parameters are used to define prognostic subgroups to determine the line of therapy and predict prognosis (WHO, 2016).

However, among all prognostic factors, the most important is the response to therapy as indicated by minimal residual disease (1) currently called measurable residual disease (MRD). MRD is the single most important prognostic factor in both pediatric (2, 3) and adult acute lymphoblastic leukemia (4–6) as well as in AML (7, 8). The impact of MRD status on survival in all types of acute leukemia is well established, and it helps in determining the line of therapy including the decision of stem cell transplantation (5, 9). Targeting MRD to prevent relapse is one of the major challenges in treatment of acute leukemia (10).

However, the impact of MRD status on therapeutic outcome is not absolute; that is, some patients with an MRD-negative status may still suffer from relapse (11).

The methodology of MRD whether molecular or by flow cytometry relies on detecting residual leukemic cells at defined time points during therapy (12, 13). Flow cytometry, the most popular and practical methodology for detection of MRD, has two limitations. First, not all the cases express a leukemia-associated immune phenotype (LAIP) that can be used for follow-up. Second, not all leukemia cells equally contribute to the proliferative status responsible for maintaining the malignant population, ultimately leading to refractoriness or relapse. Malignant cells are extremely heterogeneous (14, 15) with only a fraction, of variable size in different patients, that is responsible for initiating (16) and maintaining the leukemic population as well as resistance to therapy and later to relapse (10, 17, 18). This population is now recognized as leukemia stem cells (LSCs); it has been suggested that detection of LSCs would reduce false-negative MRD results (19).

Leukemia is now considered to be a stem cell disease with its characteristic refractory nature being blamed on a rare population of CD34+/CD38- LSCs. Furthermore, LSCs are resistant to most current therapeutic measures, which make them an important area of research as a possible target to weaken acute leukemia’s ability to relapse and remain refractory to treatment.

Many studies have documented the impact of LSC frequency at diagnosis on the therapeutic outcome and survival in acute myeloid leukemia (17–21). So, we hypothesized that LSC frequency at diagnosis may be used as a surrogate marker for MRD status on follow-up. This will give an upfront prognostic indicator that might help early planning of an appropriate therapeutic strategy.

## 2 Material and Methods

### 2.1 Patients

This study comprised 84 newly diagnosed adult AML patients including 51 men (60.7%) and 33 women (39.3%) with an age range of 18–70 with a mean of 33.4 ± 11.9 and a median of 30 years. All patients presented to the Medical Oncology outpatient department, NCI, Cairo University. The study was approved by the IRB of NCI, Cairo University, and written informed consent was obtained from all patients. The study was performed according to the requirements of the Helsinki declaration and its amendments for studies involving human beings.

All patients, except M3, received the 3 + 7 protocol.

### 2.2 Methods

Patients were diagnosed according to standard parameters including the following:

- Full history and clinical examination;
- Complete blood picture;
- Bone marrow examination;
- Cytochemistry as relevant;
- The common fusion genes for AML including t(15;17), t(8;21), and inv (16) as well as FLT-3 ITD.

### 2.3 Flow Cytometric Studies

Monoclonal antibodies (Mo Ab) with different specificities as well as the corresponding isotype controls were used. Monoclonal antibodies were obtained from BD, Franklin Lakes, New Jersey, USA; Beckman Coulter, Miami, USA; and Miltenyi Biotec, Bergisch Gladbach, Germany. Acquisition and analysis were performed on a flow cytometer (Navios Cytometer, Beckman Coulter, Miami, USA).

#### 2.3.1 Detection of Surface Markers by Direct Staining

The whole blood staining method was performed (22). In short, 10 µl of labeled Mo Ab was added to a 100-µl BM sample, incubated in the dark for 20 min, hemolyzed with a hemolyzing solution containing NH4CL, and washed with PBS then analyzed.

#### 2.3.2 Detection of Intracellular Markers

One hundred µl of whole blood was lysed using a lysis solution for 10 min. Cells were washed once and resuspended in 1 ml of PBS. A mixture of 500 µl of 4% paraformaldehyde as fixative, 500 µl of PBS, and 5 µl of Tween 20 as detergent was added to the cells and incubated for 10 min. The cells were washed and 10 µl of Mo Ab added and incubated for 30 min at 4°C. Cells were washed, resuspended in 500 µl of PBS, and analyzed. Any antigen was considered positive when ≥20% of blast cells were stained above the negative control, except for CD34, CD10, and MPO where ≥10% was considered positive (22).

#### 2.3.3 Minimal/Measurable Residual Disease Detection

A flow cytometric detection of MRD was done on EDTA bone marrow at day 14 and day 28.

After initial immunophenotyping at diagnosis, Mo Ab combinations were used to define leukemia-associated phenotypes (expressed on ≥50% of the cells). This step served to define a leukemia phenotypic fingerprint to be used in follow-up samples. At least 2 Ab combinations were used to minimize pitfalls due to phenotypic switches.

MRD studies were performed on erythrocyte-lysed whole BM. Six-color labeling was performed, and life gate analysis was used.

### 2.4 AML MRD Combination Panels

⊳ CD7/CD34/CD45/CD13/CD33/CD19
⊳ CD34/NG2/CD45/CD33/CD135/CD2
⊳ TDT/CD117/CD45/CD33/CD13/CD56
⊳ CD64/CD135/CD45/CD33/CyCD13/CyCD22
⊳ Any aberrant T-cell, B-cell, or NK marker could be included in the MRD panel at the individual case level.

Two panels were selected for each case according to the antigen expression pattern at diagnosis. The selected panels were run at diagnosis, day 14, and day 28.

### 2.5 Gating Strategy for IPT and Detection of MRD Panels

The sequential Boolean gating strategy was adopted. In AML cases, the cell population with primitive marker expression lies in a gate with low side scatter (SS) and dim CD45. Subsequently, the gated cells were analyzed for detection of various markers for initial IPT as well as for leukemia-associated immunophenotypes (LAIP). The rationale for MRD detection was to use a sequential gating strategy. Leukemic events were defined with a dot plot in a predetermined region; 500,000 CD 45+ events were acquired. At least 50 events should be present to be considered a cluster for MRD detection; back-gating was used to confirm their position in the forward scatter (**Figure S1**).

#### 2.5.1 LSC Detection

Two four-color panels were used: CD38/CD123/CD34/CD45 and CD90/CD133/CD45/CD33.

The light-scattering characteristic of AML cells was examined as forward scatter (FS) vs. side scatter (SS) in the first histogram; 50,000 CD45+ events were acquired. The cell population with primitive marker expression lay in a gate with low SS and dim CD45 in the second histogram.

Analysis of the first panel was as follows: (CD38 FITC/CD123PE/CD34ECD/CD45PE-PC5): the gated cells were analyzed for CD34 vs. CD38 in the third and CD123 vs. CD45 in the fourth histogram (**showing dim expression of CD123**). The following populations were further calculated: total CD123, CD123+/CD34-, CD34+/CD38-/CD123+, CD34+/CD38-/CD123-, CD34+/CD38+/CD123+, CD34+/CD38+/CD123-, CD34-/CD38+/CD123+, CD34-/CD38+/123-, and CD34-/CD38-/CD D123+.

Analysis of the second panel was as follows (CD90 FITC/CD133PE/CD45ECD/CD33PE-PC5): the gated cells were analyzed for CD90 vs. CD133 in the third and CD45 vs. CD33 in the fourth histogram. The following populations were further calculated: total CD133, CD133+/CD33-, total CD90, CD90+/CD33-, CD90+/CD133-/CD33-, CD133+/CD90-/CD33-, and CD90+/CD133+/CD33-.

In all, both CD45+ and CD45-ve populations were included.

The analysis, for both panels, was done at the time of diagnosis, day 14, and day 28.

The prism function of the software was used to estimate the percentage of cell populations expressing different antigen combinations. The number of potential combinations equaled the square of the number of Mo Abs in the panel. The prism processor represents data from 1, 2, 3, and 4 Ab combinations presented as evenly spaced peaks in a single-parameter graph with 16 possible combinations. Each peak represents an Ab combination or phenotype. The first peak represents negative cells, the second peak represents cells that are positive for the first parameter and negative for all other parameters, and so on, with the last peak representing cells positive for the four Abs. The height of the peak is proportional to the number of events belonging to the phenotype represented by the peak.

An example of prism analysis of both panels is shown in **Figures S2** and **S3**.

### 2.6 Statistical Methods

Statistical analysis was performed using SPSS version 21. The mean and standard deviation or the median and range were used for numerical data. Categorical data were summarized as percentages. Pearson’s correlation test was used for numerical data. Overall survival (OS) and disease-free survival (DFS) were analyzed by Kaplan–Meier curves and compared by log-rank tests. The Cox proportional hazard model was used for significant variables. All p-values are two-sided. p-values < 0.05 were considered significant.

## 3 Results

### 3.1 Patients’ Characteristics

In the current study, we evaluated LSC frequency at diagnosis in 84 newly diagnosed AML patients including 51 men (60.7%) and 33 women (39.3%) with an age range of 18–70 with a mean of 33.4 ± 11.9 and a median of 30 years. Hematological parameters are presented in **Table 1**. Normal cytogenetics were present in 61 cases (72.6%), t (8;21) in 14 (16.6%), inv 16 in 7 (8.4%), and t(15;17) in 4 (4.8%). Wild-type FLT3 was encountered in 63 and ITD in 21 cases. According to ELN risk stratification, 25 cases were in the favorable and 59 in the intermediate genetic category. Cases with t(15;17) were excluded from survival studies.

**Table 1:** Hematological parameters in 84 newly diagnosed acute myeloid leukemia cases.

Parameter	Mean ± SD	Median	Range
**TLC × 10^9^/L**	53.0 ± 60.0	30	1.8–289
**Hb gm/dl**	7.0 ± 1.9	7.0	3.3–13
**Platelets × 10^9^/L**	55.7 ± 37.3	43.9	9.0–151
**PB blasts %**	44.0 ± 29	39	0–95
**BM blasts (%)**	51.0 ± 25.0	59	21–95

### 3.2 Leukemia Stem Cell Frequency at Diagnosis

Using two simple four-color panels, we used the spike function of the software to determine the various patterns of expression of LSC marker combinations.

The leukemia stem cell subset frequency at diagnosis showed a marked variability according to different-marker(s) expression and for each marker within individual cases (**Table 2**).

**Table 2:** Leukemia stem cell frequency at diagnosis in 84 acute myeloid leukemia patients.

Leukemia stem cell marker(s)	Mean ± SD Median (range)	Leukemia stem cell marker(s)	Mean ± SDMedian (range)
**CD123**	16.9 ± 17.6	**CD34+CD38+CD123+**	2.6 ± 4.5
	9.8 (0.3–75.5)		0.9 (0.0–27.6)
**CD123+CD34-**	10.8 ± 14.9	**CD133**	20.6 ± 20.5
	4.4 (0.1–72.0)		14.1 (0.2–75.7)
**CD34-CD38+CD123-**	12.4 ± 17.6	**CD133+CD33–**	10.2 ± 13.8
	3.5 (0.0–79.0)		4.0 (0.0–67.4)
**CD34-CD38+CD123+**	3.0 ± 5.9	**CD90**	33.9 ± 28.9
	0.6 (0.0–31.0)		37.0 (0.2–92.0)
**CD34+CD38-CD123+**	1.8 ± 3.2	**CD90+CD33–**	12.6 ± 16.6
	0.7 (0.0–19.2)		3.5 (0.0–85.8)
**CD34+CD38-CD123-**	16.3 ± 19.3	**CD90+CD133–CD33–**	9.5 ± 13.2
	7.6 (0.0–79.0)		2.1 (0.0–78.4)
**CD34-CD38-CD123+**	6.4 ± 10.6	**CD133+CD90–CD33–**	6.2 ± 12.0
	2.7 (0.0–61,6)		0.7 (0.0–67.0)
**CD34+CD38+CD123-**	2.8 ± 5.2	**CD133+CD90+CD33–**	3.1 ± 6.4
	0.6 (0.0–31.3)		0.8 (0.0–35.6

No correlation was encountered between LSC frequency at diagnosis and any of the hematological parameters except for the correlation between both PB and BM blasts for CD133 (r = 0.27, p = 0.01; r = 0.36, p=0.001), CD133+/CD33- (r = 0.257, p = 0.019; r = 0.378, p = 0.001), and CD133+/CD90-/CD33- (r = .257, p = 0.019); r = 0.373, p = 0.001) respectively and the correlation of PB blasts with CD34+/CD38-/CD123+ (r = 0.315, p = 0.005) and CD34+/CD38-/CD123- (r = 0.338, p = 0.002).

### 3.3 Impact of LSC Frequency at Diagnosis on Overall Survival

The OS time for the whole group showed a mean ± SE of 6.7 ± 0.8 (CI: 5.1–8.2) and a median of 3.5 months. There was no impact of clinical or hematological parameters at diagnosis on OS in 80 adult acute myeloid leukemia patients.

**Figure 1:**
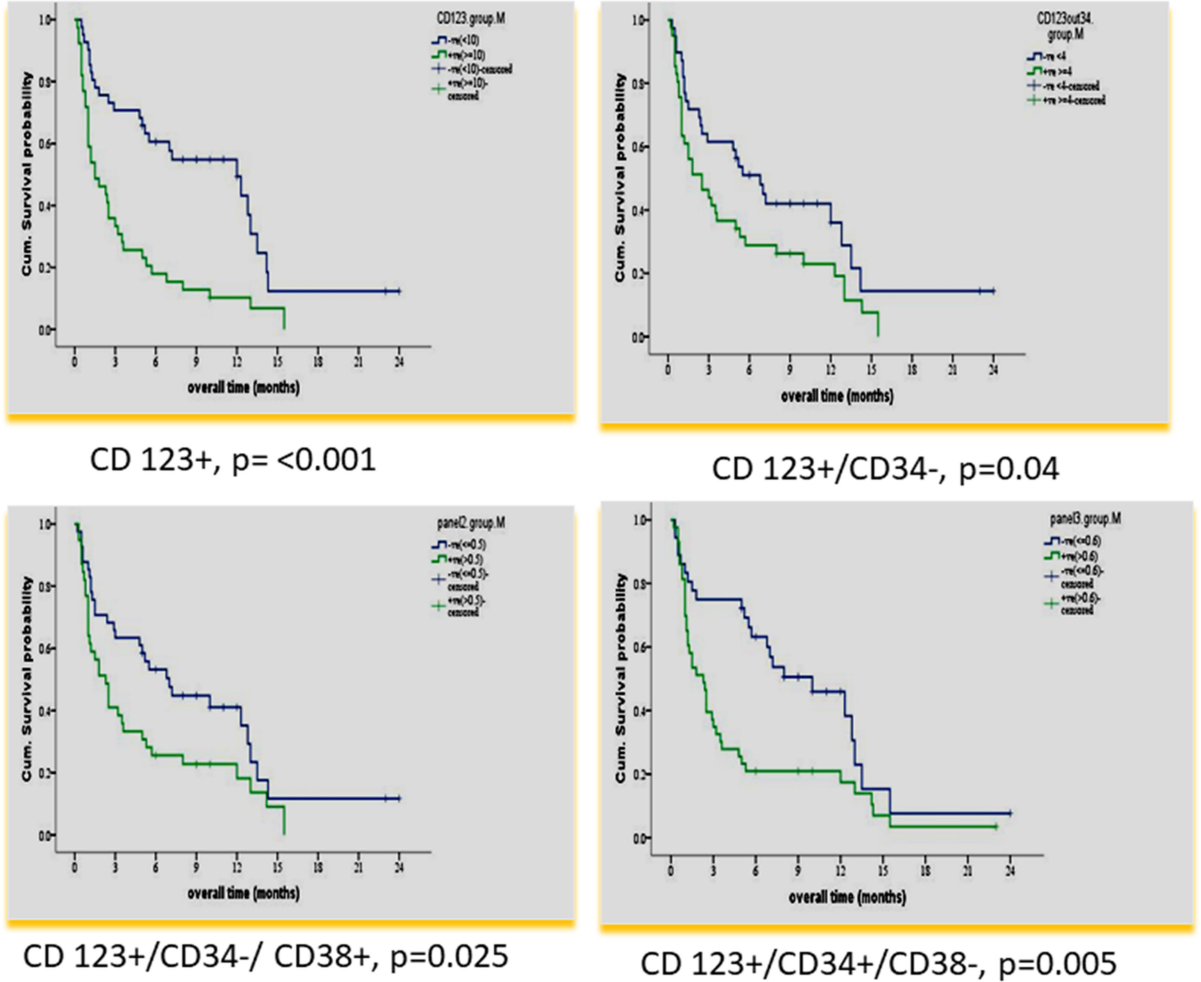
Impact of leukemia stem cell marker(s) at diagnosis on overall survival in 80 adult acute myeloid leukemia patients by using panel CD38 FITC/CD123PE/CD34ECD/CD45PE-PC5.

**Figure 2:**
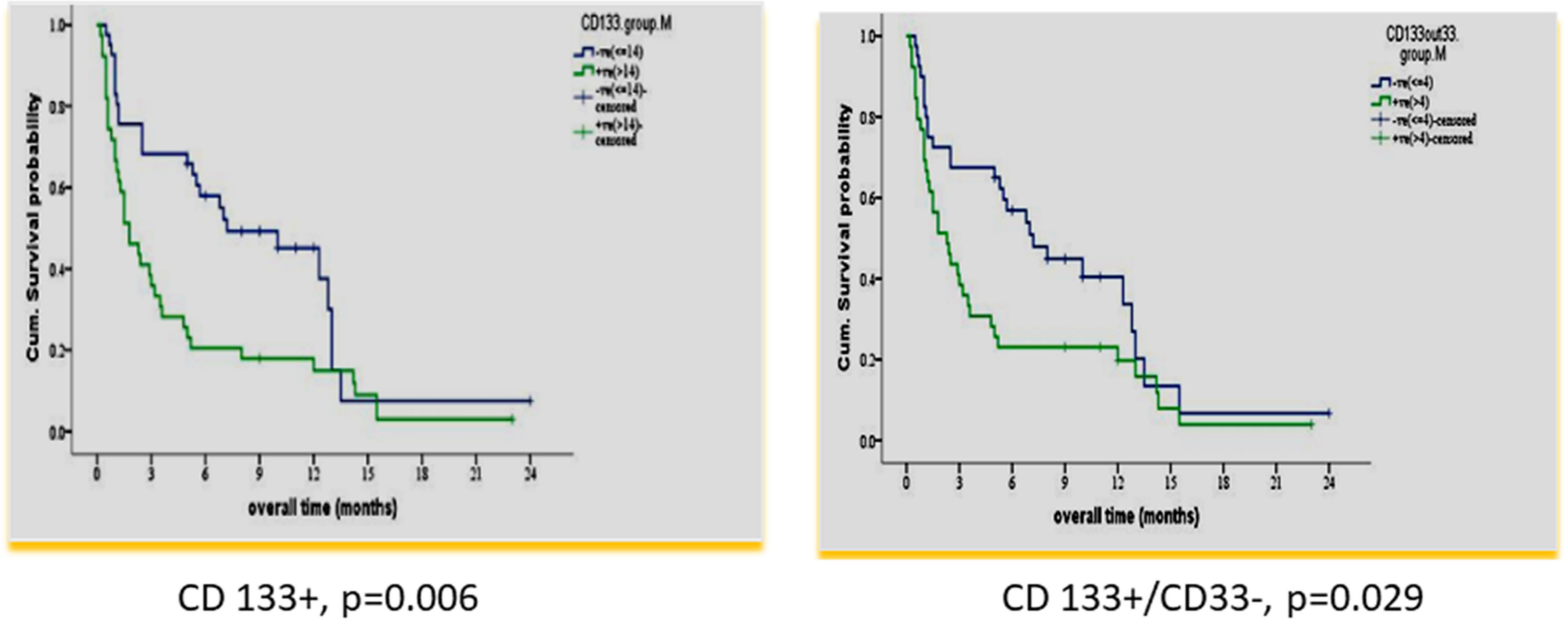
Impact of leukemia stem marker(s) on overall survival in 80 adult acute myeloid leukemia patients at diagnosis by using panel CD90 FITC/CD133PE/CD45ECD/CD33PE-PC5.

According to the median value of different markers, cases were classified into high and low expressers. Markers that showed significant impact on overall survival are presented in **Table 3** and **Figures 1**, **2**. On multivariate analysis, only CD123 (p ≤ 0.001, SE = 0.266, HR = 2.8, 95% CI = 1.74.7) and CD133+/CD33- (p = 0.017, SE = 0.263, HR = 1.9, 95% CI = 1.1–3.1) retained statistical significance.

**Table 3:** Impact of leukemia stem cell frequency (CD34/CD38/CD123) at diagnosis on overall survival in 80 acute myeloid patients.

Marker expression		N	%	Mean ± SE	Confidence interval	Median	p value
**CD123**	<10%	41	51.2	9.8 ± 1.3	7.2–12.4	12.0	<0.001
	≥10%	39	48.8	3.6 ± 0.7	2.2–4.9	1.5	
**CD123+ CD34-**	<4	39	48.7	8.7 ± 1.4	5.9–11.6	6.8	0.040
	≥4	41	51.3	5.0 ± 0.8	3.3–6.7	2.5	
**CD34-/CD38+/CD123+**	<0.5	41	51.2	8.6 ± 1.3	6.0–11.2	7.0	0.025
	≥0.5	39	48.8	4.8 ± 1.8	3.0–6.5	2.3	
**CD34+/CD38-/C123+**	<0.69	36	45	9.2 ± 1.2	6.6–11.7	10	0.005
	≥0.6	44	55	4.6 ± 0.9	2.9–6.4	2.3	
**CD133**	<14	41	51.2	8.6 ± 1.2	6.2–11	7.2	0.006
	≥14	39	48.8	4.4 ± 0.9	2.6–6.2	1.8	
**CD133+/CD33-**	<4	40	50	8.4 ± 1.1	6.0–10.7	7.0	0.029
	≥4	39	48.7	5.0 ± 0.9	3.0–6.9	2.3	

### 3.4 Impact of LSC Frequency at Diagnosis on Disease-Free Survival

The DFS time for the whole group showed a mean ± SE of 5.9 ± 0.8 (CI: 4.3–7.6) and a median of 2.3 months.

**Figure 3:**
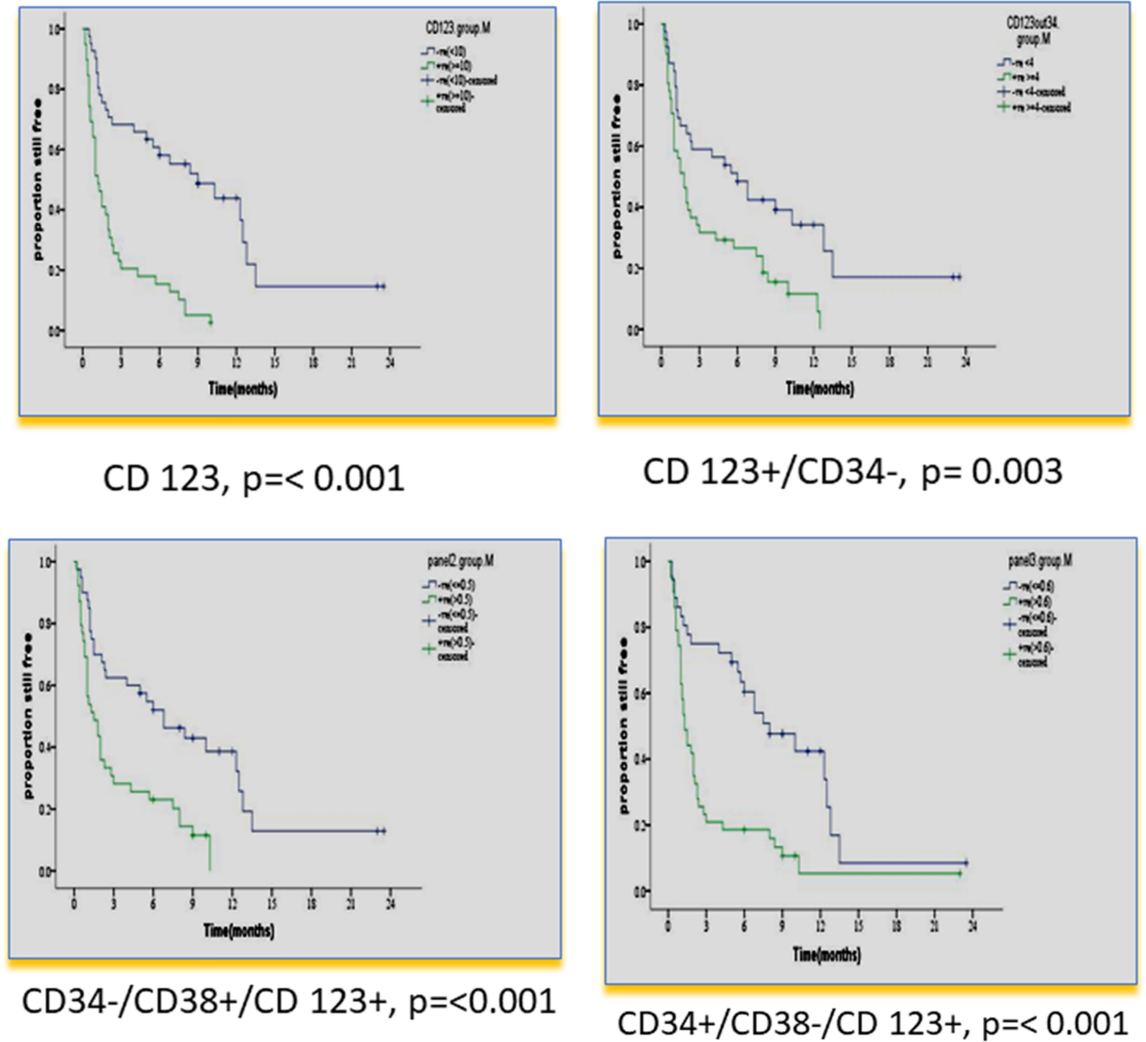
Impact of leukemia stem cell marker(s) at diagnosis on disease-free survival in 80 adult acute myeloid leukemia patients by using panel CD38 FITC/CD123PE/CD34ECD/CD45PE-PC5.

**Figure 4:**
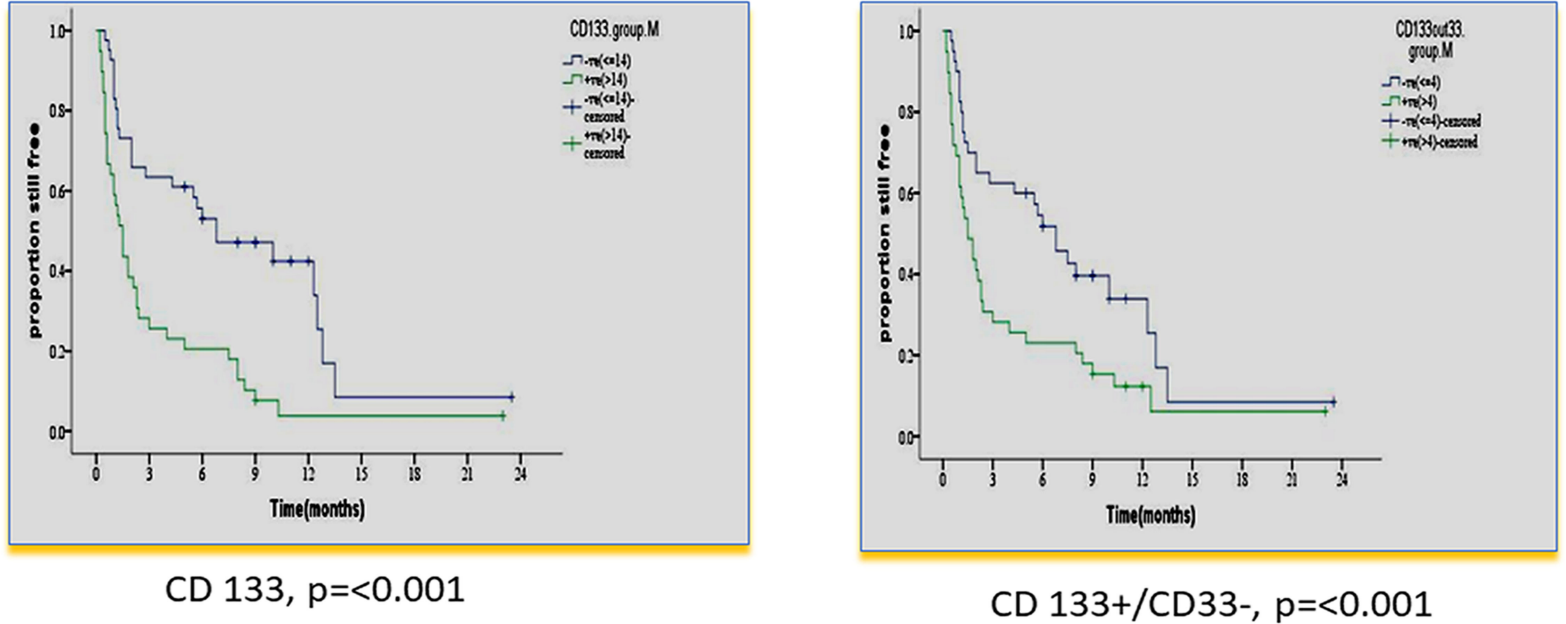
Impact of leukemia stem cell marker(s) at diagnosis on disease-free survival in 80 adult acute myeloid leukemia patients by using panel CD90 FITC/CD133PE/CD45ECD/CD33PE-PC5.

There was no impact of clinical or hematological parameters at diagnosis on DFS in 80 adult acute myeloid patients. According to the median value of different markers, cases were classified into high and low expressers. Markers that showed significant impact on DFS are presented in **Table 4** and **Figures 3**, **4**. On multivariate analysis, only CD34+/CD38-/CD123+ (p ≤ 0.001, HR 2.3, SE: 0.499, 95% CI: 2.4–17.4) and CD133 (p = 0.015, HR 2.3, SE 0.34, 95% CI: 1.2–4.4) retained statistical significance.

**Table 4:** Impact of leukemia stem cell frequency at diagnosis on disease-free survival in 80 adult acute myeloid leukemia patients.

Marker expression		N	%	Mean ± SE	Confidence interval	Median	p value
**CD123**	<10%	41	51.2	9.3 ± 1.4	6.5–12.1	9.0	<0.001
	≥10%	39	48.8	2.4 ± 0.4	1.5–3.3	1.2	
**CD123+ CD34-**	<4	39	48.7	8.5 ± 1.6	5.5–11.5	6.0	0.003
	≥4	41	51.3	3.7 ± 0.6	2.5–5.0	1.8	
**CD34-/CD38+/CD123+**	<0.5	41	51.2	8.4 ± 1.3	5.7–11.0	6.8	0.001
	≥0.5	39	48.8	3.2 ± 1.5	2.1–4.3	1.5	
**CD34+/CD38-/C123+**	<0.6	36	45	8.8 ± 1.3	6.2–11.2	8.0	<0.001
	≥0.6	44	55	.6 ± 0.9	1.8–5.3	1.3	
**CD133**	<14	41	51.2	8.2 ± 1.3	5.7–10.7	6.8	<0.001
	≥14	39	48.8	3.4 ± 0.8	1.8–4.9	1.5	
**CD133+/CD33-**	<4	40	50	7.8 ± 1.2	5.2–10.2	6.8	0.007
	≥4	39	48.7	4.2 ± 1.0	2.2–6.2	1.5	

### 3.5 Correlation Between LSC Frequency at Diagnosis and MRD

A moderate to strong positive correlation between LSC frequency at diagnosis and MRD at day 14 and day 28 was obtained for the majority of LSC markers (**Table 5**).

**Table 5:** Correlation between leukemia stem cell frequency at diagnosis in 75 adult acute myeloid leukemia patients and measurable/minimal residual disease at day 14 and day 28.

Parameter	Day 14 MRD		Day 28 MRD	
	r	p	r	p
**CD133**	**0.65**	**<0.001**	**0.65**	**<0.001**
**(CD133+/CD33-)**	0.15	0.25	**0.55**	**<0.001**
**CD123**	**0.77**	**<0.001**	**0.64**	**<0.001**
**(CD123+/CD34-)**	**0.39**	**0.001**	**0.65**	**<0.001**
**(CD34-/CD38+/CD123+)**	0.007	0.96	0.24	0.097
**(CD34+/CD38-/CD123+)**	**0.89**	**<0.001**	**0.69**	**<0.001**

MRD, measurable/minimal residual disease.

Bold denotes significance.

## 4 Discussion

### 4.1 LSC Frequency at Diagnosis

AML, like other forms of leukemia, originates from a stem cell. The lack of durable response in a high percentage of AML patients suggests that current treatments do not effectively target LSCs (23). Since it has been hypothesized that the subpopulation of chemotherapy-resistant LSCs is responsible for relapse, LSC frequency, like MRD frequency, should have a direct prognostic impact (17). New early prognostic tools based on biological analyses need to be developed.

Currently, MRD is considered an important prognostic parameter regardless of the original risk stratification at diagnosis (1–3). LSCs can also be used to monitor response to therapy (17–19). However, MRD is performed at different time points after initiating therapy while LSCs can be evaluated, as well, at diagnosis, allowing an early prognostic marker with comparable validity to MRD.

In the current work, we studied the impact of LSC frequency at diagnosis on OS and DFS and its correlation with MRD. We wanted to test the potential of LSC frequency at diagnosis to be used as a surrogate marker for MRD status allowing an early decision on the correct line of therapy. We used two simple four-color combinations. With the prism function of Coulter Navios, each gave 16 possible combinations of marker expression.

Our results showed extreme variability in the frequency of LSC subsets with expression of various markers not only between different cases but also at the individual-case level. Heterogeneity of LSCs is well documented (14, 15). This characteristic is important when LSC eradication is pursued in targeted therapy; a combination of monoclonals might be more effective than one monospecific agent.

Most previous studies used one or a few markers for LSC characterization, e.g., CD123 (24–27), CD123+/CD34+, or CD123-/CD34+% (28).

### 4.2 Correlation of LSC Frequency at Diagnosis With Hematological Parameters

In the current study, the LSC frequency at diagnosis showed no correlation with any of the hematological parameters except for blast cell percentage in both PB and BM. This is in harmony with the fact that LSCs are the initiating cells for proliferation and production of the malignant blast population (16). However, two previous studies failed to demonstrate such a correlation (29, 30). The difference may be attributed to their small sample size (29 and 30 AML patients, respectively).

### 4.3 Impact of LSC Frequency at Diagnosis on Survival

In our study, CD 123% at diagnosis had an adverse impact on OS and DFS (< p = 0.001). This result is consistent with many previous studies (24–27, 31, 32), and in agreement with a recent study, it also retained significance in multivariate analysis (33).

In our study, a higher CD 123+/CD34-% at diagnosis had a significantly adverse impact on OS and DFS (p = 0.040 and p = 0.003, respectively). To the best of our knowledge, no previous studies dealt with this population. Other studies analyzed the impact of combinations CD123+/CD34+ or CD123-/CD34+% on OS and DFS. Han et al. (28) reported a significant correlation of higher frequencies of CD34^+^/CD123^+^ blasts with shorter time to relapse in the univariate Cox proportional hazard model (p = 0.037).

In our study, the CD34+/CD38- population was dissected according to CD123 expression. Only the CD123+ subpopulation was significantly associated with adverse impacts on OS and DFS (p = 0.005 and p ≤ 0.001), respectively. This emphasizes the prognostic value of CD123 expression. Vergez et al. (26) reported that a cutoff of 1% CD34+CD38^low/-^CD123+ cells negatively affected disease-free survival and was strongly associated with early relapse.

Using variable cutoffs of 5%, 10%, and 20%, Das et al. (34) reported the expression of CD123 in 75.6%, 66.2%, and 50% of AML. They also reported that CD123 expression at diagnosis was associated with post-induction MRD-positive status (*p* = .001). Lamble et al. (33, 35) reported the association of high CD123 expression with significantly higher RR (53% vs. 39%, p < 0.001), lower EFS (49% vs. 69%, p < 0.001), and lower OS (32% vs. 50%, p < 0.001). They also reported that CD123 expression was independently associated with worse OS (HR 1.54, 95% CI 1.21–1.96, p < 0.001).

In our study, there was a significant association of the higher expression of leukemia stem cell markers: CD 133 and CD133+/CD33-% at diagnosis, with shorter OS and DFS (p = 0.006 and p ≤ 0.001 and p = 0.029 and p = 0.007). These results are consistent with some previous studies (25, 36). However, this could not be detected in other studies (37, 38). The difference may be attributed to different protocols of therapy in their study.

In our study, multivariate analysis established LSC markers CD123 (HR 2.8 and p ≤ 0.001) and CD133% (HR 1.9 and p = 0.017) at diagnosis as independent prognostic parameters for OS and DFS. This is consistent with other studies for CD123 (39) and CD 133 (25, 40).

### 4.4 Correlation of LSC Frequency at Diagnosis With MRD

Our data did show that a high level of LSC markers CD123, CD123+/CD34-, CD34-/CD38+/CD123+, CD34+/CD38-/CD123+, CD133, and/or CD133+/CD33-% at diagnosis was predictive of an increased risk of positive MRD. This finding may have important implications for early therapeutic intervention because this phenotype can be routinely established within 1 day. Hence, the assessment of the level of LSC markers at diagnosis could help clinicians to quickly identify high-risk patients and thereby improve the response rate by using different therapeutic strategies.

In this study, a moderate to strong positive correlation between LSC frequency at diagnosis and MRD at day 14 and day 28 was obtained for the majority of LSC markers (**Table 5**). Several studies used LSC markers in parallel with standard MRD panels in a follow-up of AML (8, 19, 33, 41–43). Two previous studies addressed the association of LSC frequency at diagnosis with MRD (28, 34). However, they only tested one population (CD123+/CD34+ and CD123+, respectively). To the best of our knowledge, this is the first study addressing the potential of LSC frequency at diagnosis for prediction of MRD status on follow-up by using several-marker combinations. Thus, apart from being associated with OS and DFS, the LSC frequency at diagnosis can also predict, upfront, which patients will have an MRD+ status on follow-up. Taking into consideration the prognostic value of MRD and that of LSC frequency at diagnosis, we have a strong prognostic parameter at diagnosis that would allow for an early decision on the appropriate therapeutic approach.

## 5 Conclusion

AML cases show a marked variability in the number and phenotype of LSCs, which have to be taken into consideration when designing targeted therapy. Our work further established the prognostic value of LSC frequency at diagnosis and emphasized the independent prognostic role of some LSC markers, namely, CD123 and CD133. We report the value of LSC frequency at diagnosis, detected by several-marker combinations, to predict the MRD status on follow-up; this further potentiates the value of the LSC frequency at diagnosis as an early prognostic parameter allowing early therapeutic decisions, which might help to improve the outcome in AML.

## Data Availability Statement

The original contributions presented in the study are included in the article/**Supplementary Material**. Further inquiries can be directed to the corresponding author.

## Ethics Statement

The studies involving human participants were reviewed and approved by the NCI, Cairo University Institutional Review Board. The patients/participants provided their written informed consent to participate in this study.

## Author Contributions

AK conceived of and designed the study, analyzed the data, and wrote the manuscript. NE conceived of and designed the study and supervised the practical work. EK designed the study, designed the experiments, analyzed the data, and edited the manuscript. MH and RO performed the research, analyzed the data, and edited the manuscript. MS was responsible for the clinical data and follow-up of patients. All authors contributed to the article and approved the submitted version.

## Funding

This work was funded by Cairo University.

## Conflict of Interest

The authors declare that the research was conducted in the absence of any commercial or financial relationships that could be construed as a potential conflict of interest.

## Publisher’s Note

All claims expressed in this article are solely those of the authors and do not necessarily represent those of their affiliated organizations, or those of the publisher, the editors and the reviewers. Any product that may be evaluated in this article, or claim that may be made by its manufacturer, is not guaranteed or endorsed by the publisher.
