# Supplementary figures and images for: Leukemia Stem Cell Frequency at Diagnosis Correlates With Measurable/Minimal Residual Disease and Impacts Survival in Adult Acute Myeloid Leukemia
Source: Front Oncol. 2022 Apr 8;12:867684. doi: 10.3389/fonc.2022.867684 (PMC9069678; doi:10.3389/fonc.2022.867684)

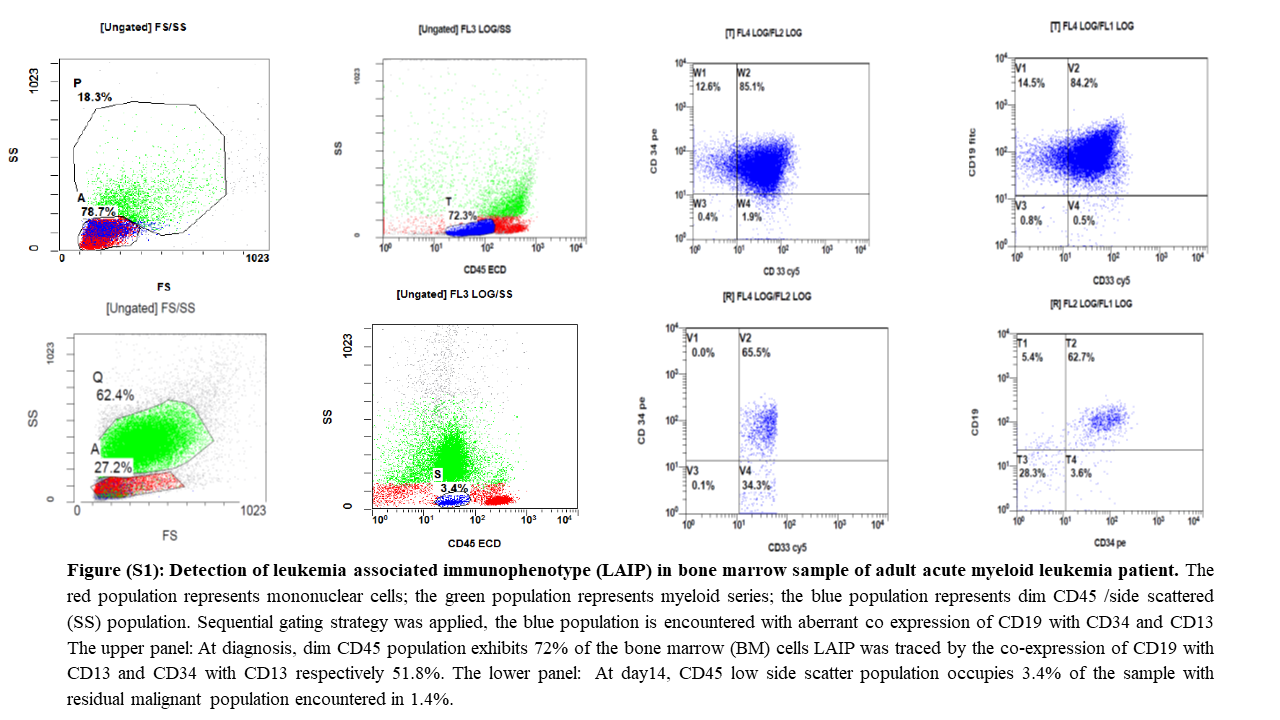

Supplement: Supplementary file 1 [file Image_1.tif]

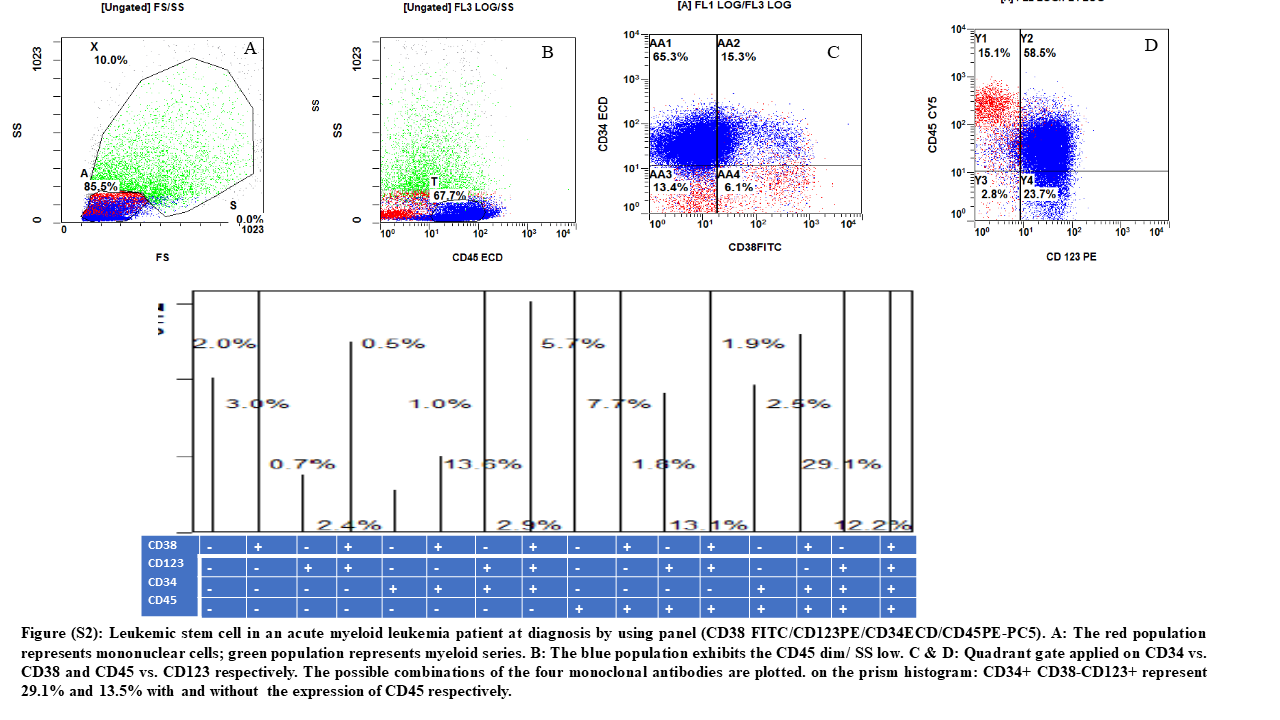

Supplement: Supplementary file 2 [file Image_2.tif]

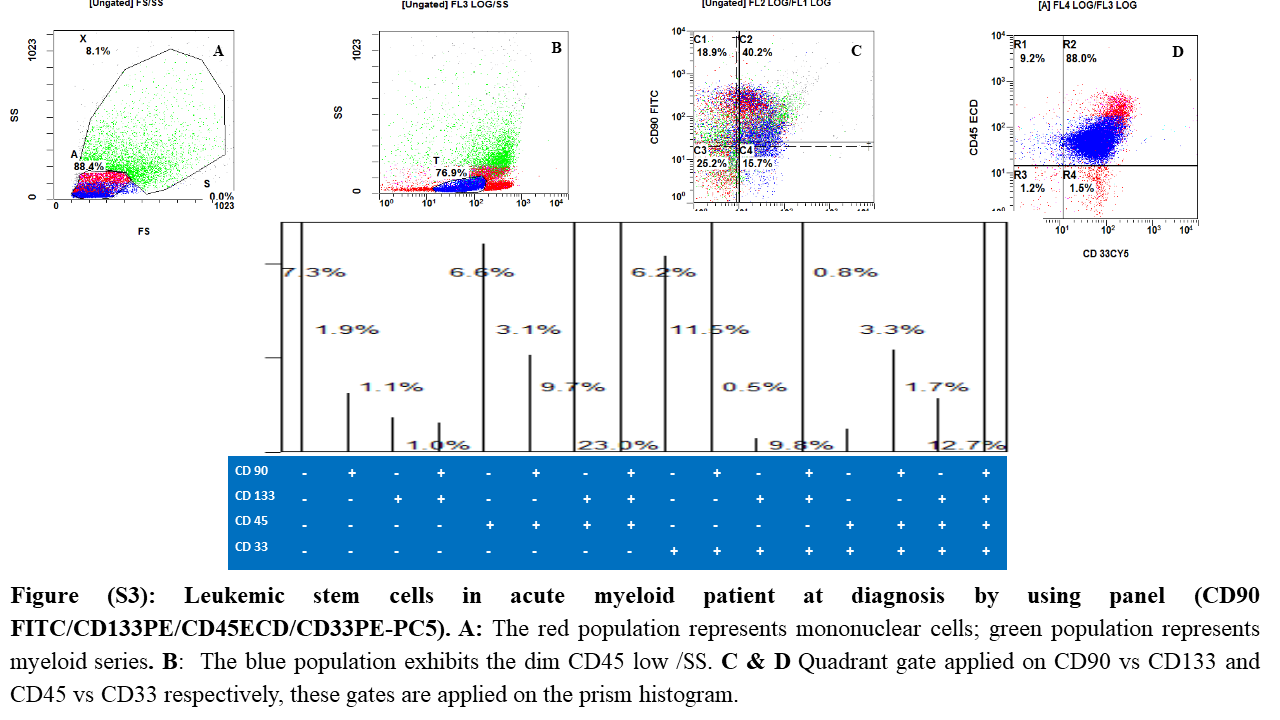

Supplement: Supplementary file 3 [file Image_3.tif]
